# Knowledge, perception, and practice related to sodium intake among Malaysian adults: findings from the Malaysian Community Salt Study (MyCoSS)

**DOI:** 10.1186/s41043-021-00231-4

**Published:** 2021-05-31

**Authors:** Siew Man Cheong, Rashidah Ambak, Fatimah Othman, Feng J. He, Ruhaya Salleh, Syafinaz Mohd Sallehudin, Lalitha Palaniveloo, Shubash Shander Ganapathy

**Affiliations:** 1 Institute for Public Health, National Institutes of Health, Ministry of Health Malaysia, Selangor Shah Alam, Malaysia; 2 Centre for Environmental and Preventive, Medicine Wolfson Institute of Preventive Medicine, Queen Mary University of London, London, UK

**Keywords:** Urinary sodium excretion, Salt knowledge, Sodium intake, Self-perceived salt intake

## Abstract

**Background:**

Excessive intake of sodium is a major public health concern. Information on knowledge, perception, and practice (KPP) related to sodium intake in Malaysia is important for the development of an effective salt reduction strategy. This study aimed to investigate the KPP related to sodium intake among Malaysian adults and to determine associations between KPP and dietary sodium intake.

**Methods:**

Data were obtained from Malaysian Community Salt Survey (MyCoSS) which is a nationally representative survey with proportionate stratified cluster sampling design. A pre-tested face-to-face questionnaire was used to collect information on socio-demographic background, and questions from the World Health Organization/Pan American Health Organization were adapted to assess the KPP related to sodium intake. Dietary sodium intake was determined using single 24-h urinary sodium excretion. Respondents were categorized into two categories: normal dietary sodium intake (< 2000 mg) and excessive dietary sodium intake (≥ 2000 mg). Out of 1440 respondents that were selected to participate, 1047 respondents completed the questionnaire and 798 of them provided valid urine samples. Factors associated with excessive dietary sodium intake were analyzed using complex sample logistic regression analysis.

**Results:**

Majority of the respondents knew that excessive sodium intake could cause health problems (86.2%) and more than half of them (61.8%) perceived that they consume just the right amount of sodium. Overall, complex sample logistic regression analysis revealed that excessive dietary sodium intake was not significantly associated with KPP related to sodium intake among respondents (*P* > 0.05).

**Conclusion:**

The absence of significant associations between KPP and excessive dietary sodium intake suggests that salt reduction strategies should focus on sodium reduction education includes measuring actual dietary sodium intake and educating the public about the source of sodium. In addition, the relationship between the authority and food industry in food reformulation needs to be strengthened for effective dietary sodium reduction in Malaysia.

## Background

Excessive intake of sodium is a major public health concern. High sodium consumption is an important risk factor for non-communicable diseases, including hypertension, stroke, heart disease, and stomach cancer [1, 2]. The World Health Organization (WHO) has recommended no more than 2000 mg per day of dietary sodium intake for adults [3] to maintain optimum health. A fairly recent study among Ministry of Health staff estimated that Malay working adults generally consume 2860 mg of sodium per day which is above the WHO recommendation, with most of their dietary sodium coming from composite foods and sauces [4]. However, this sodium consumption is lower than reports from other countries with diverse diets, such as Korea (4349 mg per day) [5], China (5560 mg per day) [6], USA (3608 mg per day) [7], and Iran (3808 mg per day) [8].

A systematic review which was published in 2015 indicated that a total of 75 countries have a national salt reduction strategy and most of the countries focus on consumer education and product reformulation [9]. Similarly, the Ministry of Health Malaysia, aware of the negative health impact of excessive sodium intake, initiated a national salt reduction strategy in 2015 to raise awareness and knowledge of salt reduction through campaigns, mass media, and social marketing. The salt reduction strategies in Malaysia include monitoring of sodium intake among the Malaysian population, education on the relationship between sodium intake and diseases and sources of sodium, and continuing the current partnership with food industries for food product reformulation [10]. Individuals’ knowledge and behavior influenced their food choices because individual’s salt knowledge, perception towards salt, and salt taste beliefs were known to be mediating factors of salt usage [11]. Another study revealed that knowledge, perception, and practices (KPP) is a useful tool targeted as a baseline assessment for intervention at population level [12].

The implementation of these salt reduction strategies were done by The Ministry of Health Malaysia. Although there was a local study conducted to determine the amount of dietary sodium intake and dietary sources of sodium among working adults in the Ministry of Health Malaysia, there is still lack of national representative data on dietary sodium intake and its associated factors [4]. Therefore, this study aimed to investigate the knowledge, perception, and practices (KPP) related to sodium intake among Malaysian adults and determine the associations between KPP and sodium intake level. Information on KPP related to sodium intake among Malaysian population provide useful baseline data for policy-makers to implement more effective salt reduction strategies as well as to evaluate the effectiveness of salt reduction.

## Methods

**Fig. 1:**
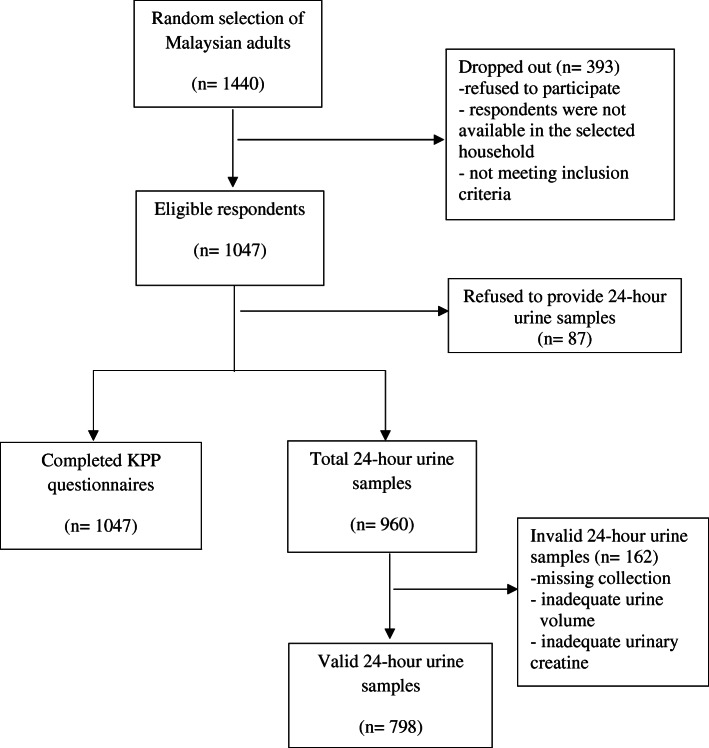
Study recruitment flow chart

Data from this study were obtained from the Malaysian Community Salt Survey (MyCoSS). MyCoSS is a population representative household survey conducted among Malaysian adults aged 18 years and above. The survey was based on proportionate stratified cluster sampling design by states based on urban and rural Enumeration Blocks (EBs). First stage sampling unit was 80,000 EBs in Malaysia. Living Quarter (LQ) within the EBs was the second stage sampling. Each EB consists of 80 to 120 LQs. A total of 1440 LQs were randomly selected. Only one Malaysian adult aged 18 years and above was recruited randomly into this study from each selected LQ. Face-to-face interview sessions were conducted by trained research team members to collect information on socio-demographic background and KPP related to sodium intake using mobile devices. Interviewers entered the answers obtained from the respondents into the questionnaire system application, IKU Survey Creation System. Completed questionnaires were sent to Survey Creation System server. Data in the server were downloaded by the data management team in Institute for Public Health. Out of 1440 respondents were selected to participate in MyCoss, 1047 agreed to participate in this study and completed the KPP questionnaire with the response rate of 72.7%. However, among 1047 respondents, only 960 of them provided 24-h urine samples. A total of 798 of them provided valid urine sample with adequate volume (at least 500 ml) and adequate urinary creatinine as well as without reported missing collection within 24 h. The study recruitment flow-chart is shown in Fig. 1.

### Socio-demographic variables

The socio-demographic variables collected were age, sex, ethnicity, marital status, education achievement, and household income. The age of the respondents was determined by their year of birth and then age was categorized into five categories: less than 35 years old, 35–44 years old, 45–54 years old, 55–64 years old, and 65 years old and above. Ethnicity was categorized to Malay, Chinese, Indian, and Indigenous people from the states of Sabah, Sarawak, and states in Peninsular Malaysia.

### KPP questions related to sodium intake

The English version of the KPP questionnaire was adapted from the World Health Organization/Pan American Health Organization protocol for population level sodium determination [13]. The questionnaire was translated into the Malay language using forward-backward translation. The content validity of the KPP questions was established with expert panel consensus, and then pre-tested among adults with employment. The final version of the revised KPP questions was used in this study.

Only three out of the eight questions in the KPP questionnaire were used for statistical analysis. Respondents were asked whether they think that a high salt diet could cause a serious health problem. There is no scoring for categorization of knowledge. They were coded as having knowledge if they answered “yes” and coded as do not have knowledge if they answered “no” or “do not know” for the knowledge question. The respondents were also assessed on their perception to sodium intake using a multi-choice question: *How much salt do you think you consume?* Six options were available for this question, which were “far too much,” “too much,” “just the right amount,” “too little, “far too little,” and “do not know.” Then, the responses were grouped into “too much,” “just the right amount,” and “too little.” The practice of controlling their sodium intake was assessed using one question: *Do you do anything on a regular basis to control your salt/sodium intake?* They were coded as having sodium control practice if they answered “yes” and coded as do not have sodium control practice if they answered “no” and do not know.” The questions and coded responses are listed in Table 4 in Appendix.

### 24-h sodium intake

Dietary sodium intake was assessed using single 24-h urinary sodium excretion from eligible respondents. Instructions to collect urine sample were given by the field researchers. Indirect ion-selective electrode method was used for sodium analysis. Sodium samples with a volume less than 500 ml and incomplete 24-h urine sample (urinary creatinine as indicator) were excluded from the sodium analysis. In addition, 24-h urine samples were excluded if the 24-h urinary creatinine < 6 mmol/day for males and 4 < mmol/day for females [14]. The 24-h sodium excretion value was calculated in milligrams (mg) per day. Then, the data on 24-h urinary sodium excretion was categorized into normal dietary sodium intake (< 2000 mg sodium) and excessive dietary sodium intake (≥ 2000 mg sodium) [3].

### Data analysis

Data analyses were conducted using SPSS version 22 (SPSS IBM, New York, USA). Weight factors were applied for the complex study design and non-response rate. The complex sample design and weights were taken into account in all statistical analyses. Descriptive statistics and ANOVA tests were used to describe the socio-demographic variables and KPP related to sodium intake. Factors associated with excessive dietary sodium intake were analyzed using complex sample logistic regression analysis controlling for socio-demographic variables. All statistical analyses were described using 95% confidence intervals at a statistical significance level of *P* < 0.05.

## Results

Table 1 presents the socio-demographic characteristics of the respondents. About 60% of the respondents were 45 years old and above. A majority of the respondents were female, Malay ethnicity, and married. Over half of the respondents achieved secondary education level. Based on 24-h urinary sodium excretion, 78.6% (95% CI 74.6–82.1) of them consumed sodium exceeding the daily recommended intake by the WHO which was less than 2000 mg per day [3].

**Table 1:** Socio-demographic characteristics of the respondents

Characteristics	*n*	% (95% CI)
**Age** Mean 48.81 (95% CI 47.03–50.60)		
< 35 years	232	22.0 (18.1–26.4)
35–44 years	176	17.2 (13.5–21.6)
45−54 years	216	20.3 (17.1–23.9)
55–64 years	244	23.3 (19.6–27.5)
≥ 65 years	179	17.2 (13.8–21.3)
**Sex**		
Male	428	50.2 (45.6–54.7)
Female	619	49.8 (45.3–54.4)
**Ethnicity**		
Malay	662	67.2 (58.1–75.2)
Chinese	116	11.6 (7.2–18.1)
Indian	63	9.4 (5.8–14.8)
Indigenous people	206	11.8 (6.7–20.0)
**Marital status**		
Single	133	13.0 (10.1–1635)
Married	760	75.5 (71.7–78.9)
Separated/widowed	153	11.6 (8.9−14.9)
**Education level**		
Without formal education	96	5.5 (3.8–7.9)
Primary	220	18.7 (15.1–22.9)
Secondary	503	51.3 (47.4–55.2)
Tertiary	228	24.5 (20.3–29.2)
**Household income** Mean RM 3292.44 (95% CI 2840.57, 3744.31)		
<RM 1000	324	24.7 (20.5–29.4)
RM 1000–1999	203	18.1 (15.1–21.6)
RM 2000–2999	174	16.1 (12.6–20.4)
RM 3000–3999	125	13.8 (10.4–18.1)
≥ RM 4000	221	27.3 (21.4–34.2)
**Employment status**		
Employed	317	32.2 (27.2–37.7)
Self-employed	232	21.9 (18.1–26.2)
Housewives	298	25.6 (21.9–29.6)
Unemployed/students/others	200	20.3 (17.3–23.7)
**24-h urinary sodium excretion** Mean 3167 mg (95% CI 2987, 3346)		
Normal (< 2000 mg)	207	21.4 (17.9–25.4)
Excessive (≥ 2000 mg)	591	78.6 (74.6–82.1)

Table 2 shows the KPP related to sodium intake among the respondents. A total of 86.2% (95% CI 82.7–89.1) of the respondents reported they have the knowledge that high dietary salt intake could cause health problems. More than half of the respondents (62.6%, 95% CI 58.3–66.7) perceived that they consumed the right amount of salt with 55.4% (95% CI 50.6–60.1) reported that they actively controlled their dietary sodium intake. Table 3 presents the factors associated with high dietary sodium intake among Malaysian adults. Complex sample logistic regression analyses indicated that all KPP factors were not significantly (*p* > 0.05) associated with excessive dietary sodium intake.

**Table 2:** Knowledge, perception, and practice related to sodium intake among respondents

Question	*n*	% (95% CI)^a^	Mean 24-h urinary sodium excretion^b^	*F*
**Do you think that a high salt diet could cause a serious health problem?**				*P* = 0.688
Yes	861	86.2 (82.7–89.1)	3008.79 ± 1387.80	
No/do not know	170	13.8 (10.9–17.3)	2953.61 ± 1583.06	
**How much salt do you think you consume?**				*P* = 0.530
Too much	139	14.9 (11.4–19.2)	3115.01 ± 1375.75	
Just the right amount	613	62.6 (58.3–66.7)	2985.25 ± 1407.90	
Too little	251	22.5 (19.0–26.5)	2924.24 ± 1416.89	
**Do you do anything on a regular basis to control your salt/sodium intake?**				*P* = 0.420
Yes	601	55.4 (50.6–60.1)	2962.23 ± 1388.27	
No/do not know	429	44.6 (39.9–49.4)	3044.57 ± 1444.41	

^a^Frequency test

^b^One-way ANOVA tests

**Table 3:** Association between KPP related to excessive 24-hour urinary sodium excretion

KPP	Crude OR^**a**^	***p***	Adjusted OR^**b**^	***p***
Age				
< 35 years	2.001 (1.259-3.180)	0.023	1.589 (0.765-3.298)	0.211
35-44 years	2.257 (1.120-4.545)	0.011	1.919 (0.793-4.646)	0.146
45-54 years	2.654 (1.260-5.589)	0.006	1.774 (0.924-3.403)	0.084
55-64 years	2.377 (1.291-4.378)	0.138	1.288 (0.612-2.708)	0.501
≥65 years	1.00 (ref.)		1.00 (ref.)	
Sex				
Female	0.497 (0.292-0.845)	0.010	0.375 (0.218-0.646)	0.01*
Male	1.00 (ref.)		1.00 (ref.)	
Ethnicity				
Chinese	0.736 (0.360-1.505)	0.397	0.801 (0.395-1.625)	0.534
Indian	0.512 (0.210-1.249)	0.139	0.634 (0.262-1.534)	0.309
Indigenous people	0.728 (0.443-1.197)	0.208	0.824 (0.460-1.473)	0.509
Malay	1.00 (ref.)		1.00 (ref.)	
Marital status				
Married	0.921 (0.446-1.905)	0.823	0.837 (0.437-1.604)	0.588
Separated/widowed	0.331 (0.130-0.845)	0.021	0.450 (0.178-1.135)	0.090
Never married	1.00 (ref.)		1.00 (ref.)	
Household income				
< RM 1000	0.481 (0.264-0.877)	0.018	0.945 (0.450-1.984)	0.880
RM 1000-1999	0.859 (0.409-1.808)	0.687	1.176 (0.563-2.457)	0.662
RM 2000-2999	0.585 (0.266-1.286)	0.180	0.699 (0.330-1.479)	0.345
RM 3000-3999	0.579 (0.224-1.497)	0.256	0.706 (0.303-1.644)	0.415
≥ RM 4000	1.00 (ref.)		1.00 (ref.)	
Employment status				
Self-employed	1.068 (0.550-2.076)	0.844	1.183 (0.558-2.510)	0.657
Housewives	0.549 (0.307-0.982)	0.043	1.168 (0.579-2.356)	0.644
Unemployed/students/others	0.400 (0.216-0.740)	0.004	0.489 (0.237-1.012)	0.054
Employed	1.00 (ref.)		1.0 (ref.)	
Do you think that a high salt diet could cause a serious health problem?				
Yes	1.316 (0.820-2.111)	0.251	1.622 (0.905-2.906)	0.103
No/ do not know	1.00 (ref.)		1.00 (ref.)	
Self-perceived salt intake				
Too much	1.129 (0.532-2.396)	0.749	-	
Too little	0.735 (0.421-1.283)	0.275		
Just the right amount	1.00 (ref.)			
Do you do anything on a regular basis to control your salt/sodium intake?				
Yes	1.372 (0.866-2.176)	0.176	1.329 (0.816-2.163)	0.250
No/ do not know	1.00 (ref.)		1.00 (ref.)	

Dependent variable: 24-h urinary sodium excretion (reference category = < 2000 mg)

*significant at *P* < 0.05

^a^Only *P* ≤ 0.25 will be recruited into adjusted model in complex sample logistic regression

^b^Controlling variables for adjusted model in complex sample logistic regression: age, sex, ethnicity, marital status, education achievement, household income, and employment status

## Discussion

This study found that most of the adult population in Malaysia were aware that eating too much sodium is harmful to their health; however, they did not realize that their sodium intake has been very high. Almost three quarters of the respondents consumed dietary sodium more than 2000 mg in a day but only 14.9% of them perceived that they consumed too much salt. Underestimation of own sodium intake is consistently reported in other population studies [15, 16]. This may be based on lack of knowledge of dietary sodium intake recommendation. Another possible reason may be a lack of awareness on how widespread sodium is in everyday food items, such as bread, cereal products, biscuits, and composite foods [15, 17]. A cohort study has been conducted in several countries found that although the public has misperceptions regarding their sodium intake, they still wanted to know more about sodium and the main sources of sodium in their diet [18]. Therefore, government is suggested to emphasis on salt reduction campaign and to educate the public what are the sources of dietary salt in order to increase their awareness and knowledge related to salt intake.

Our findings showed that the KPP related to sodium intake was not significantly associated with excessive dietary sodium intake (≥ 2000 mg). Most Malaysian adults consumed high dietary sodium despite claiming to know about the negative effects of high salt intake. Since most respondents also perceived their sodium intake to be “the right amount” (the perception variable), even among the 55% who actively control their sodium, it clearly reveals that the majority are not aware of their actual consumption, nor the recommended amount to avoid health problems.

These findings are comparable to one study done in Australia using the same questionnaire [16]. It also found the absence of association KPP related to sodium with 24-h urinary sodium excretion. In contrast, another study in Malaysia reported a significantly positive association between perception of sodium intake and 24-h sodium excretion, i.e., respondents who perceived their sodium intake to be high were more likely to have higher 24-h sodium excretion [19]. In that study, however, respondents comprised of Malay adults working at the Ministry of Health Malaysia. Thus, it was possible that those respondents were more aware, and accurate, about their own sodium intake compared to the general public because of more frequent exposure to healthy eating information at the workplace. An evaluation study found a significant improvement in knowledge, attitude, and practice related to salt intake among South Africans after implementation of salt reduction mass media campaign [20]. By extension, this suggests that frequent exposure to such information may also enable the public to achieve a similar level of awareness. In this regard, our study did not explore respondents’ knowledge of common foods and condiments with high sodium content, for example soy sauce, condiments, cream crackers, anchovy, and bread [4]. Poor knowledge regarding sources of dietary sodium among Malaysians has been reported [21]. Thus, we suggest that there is a need to educate the public at national level about recommended dietary sodium intake and, more importantly, about the sources of sodium in the local diet in order to raise public interest and motivation to engage in salt reduction.

The Ministry of Health Malaysia initiated its Salt Reduction Strategy in 2015. Enhancing knowledge about the relationship between sodium and non-communicable diseases through health education and communication is one of the main strategies for Malaysia [10]. This study suggests that more frequent exposure to salt reduction information may enable the public to achieve a similar level of awareness as the staff in the Ministry of Health Malaysia. Equally important is informing the public about sources and levels of sodium and enabling them to choose lower sodium options. Furthermore, it is vital that the food industry makes a gradual and sustained reduction in the amount of salt added to all of their food products and clearly labels the sodium/salt content of food to make it easier for consumers to choose lower sodium/salt options. Gradual salt reduction in the main food sources of sodium in Malaysia such as sauces or seasoning, condiments, meat products, instant noodles, and bread [4] by food reformulation is one of the cost-effective ways to reduce dietary sodium intake at national level. In addition, the development of a baseline sodium content for food sold in Malaysia is necessary to evaluate the effort of food reformulation by the food industry [22].

This is the first study to investigate the KPP related to sodium intake using a nationally representative sample compared to other previous studies in Malaysia. The findings of this study will be useful to the policy-makers and program managers to combat the high prevalence of non-communicable diseases, especially hypertension in Malaysia. However, this study has some limitations. The findings from one 24-h urinary sodium excretion did not reflect the habitual intake of sodium of the respondents because we only generated 1-day dietary sodium intake through single 24-h urine sample. We recommend future research to explore the association of KPP with habitual sodium intake using food frequency questionnaire or multiple-day 24-h urine samples instead of 1-day sodium consumption.

## Conclusion

Although Malaysian adults were aware of the negative impacts of excessive sodium intake, Malaysian adults still consumed high sodium. The absence of association between KPP and high dietary sodium intake is a concern. Education on dietary sodium/salt for example salt reduction campaign in mass media or social network aim to measure the public’s actual dietary sodium intake and to educate the sodium from foods may be an effective way in reducing sodium intake among the Malaysian adults. In addition, strengthen the relationship between the authority and food industry in food reformulation is also suggested to be more effective in reducing sodium intake among the Malaysian population [23].

## Data Availability

The datasets used and/or analyzed during the current study are available from the corresponding author on reasonable requests.

## Appendix

**Table 4:** KPP questions used for statistical analysis

Question	Responses
Do you think that a high salt diet could cause a serious health	Yes
	No
	Do not know
How much salt do you think you consume?	Far too much
	Too much
	Just the right amount
	Too little
	Far too little
	Do not know
Do you do anything on a regular basis to control your salt or sodium intake?	Yes
	No
	Do not know

## Abbreviations

EB: Enumeration blocks
LQ: Living quarter
MyCoSS: Malaysian Community Salt Survey
KPP: Knowledge, Perception, and Practice
WHO: World Health Organization
